# Neurochemical and Neuroanatomical Plasticity Following Memory Training and Yoga Interventions in Older Adults with Mild Cognitive Impairment

**DOI:** 10.3389/fnagi.2016.00277

**Published:** 2016-11-21

**Authors:** Hongyu Yang, Amber M. Leaver, Prabha Siddarth, Pattharee Paholpak, Linda Ercoli, Natalie M. St. Cyr, Harris A. Eyre, Katherine L. Narr, Dharma S. Khalsa, Helen Lavretsky

**Affiliations:** ^1^Semel Institute for Neuroscience and Human Behavior, University of California, Los Angeles (UCLA)Los Angeles, CA, USA; ^2^Ahmanson-Lovelace Brain Mapping Center, Department of Neurology, University of California, Los Angeles (UCLA)Los Angeles, CA, USA; ^3^Department of Psychiatry, Faculty of Medicine, Khon Kaen UniversityKhon Kaen, Thailand; ^4^Discipline of Psychiatry, University of AdelaideAdelaide, SA, Australia; ^5^IMPACT SRC, School of Medicine, Deakin UniversityGeelong, VIC, Australia; ^6^Department of Psychiatry, University of MelbourneMelbourne, VIC, Australia; ^7^Alzheimer’s Research and Prevention FoundationTucson, AZ, USA

**Keywords:** proton magnetic resonance spectroscopy, structural magnetic resonance imaging, memory enhancement training, yogic meditation, dorsal anterior cingulate cortex, hippocampus

## Abstract

Behavioral interventions are becoming increasingly popular approaches to ameliorate age-related cognitive decline, but their underlying neurobiological mechanisms and clinical efficiency have not been fully elucidated. The present study explored brain plasticity associated with two behavioral interventions, memory enhancement training (MET) and a mind-body practice (yogic meditation), in healthy seniors with mild cognitive impairment (MCI) using structural magnetic resonance imaging (s-MRI) and proton magnetic resonance spectroscopy (^1^H-MRS). Senior participants (age ≥55 years) with MCI were randomized to the MET or yogic meditation interventions. For both interventions, participants completed either MET training or Kundalini Yoga (KY) for 60-min sessions over 12 weeks, with 12-min daily homework assignments. Gray matter volume and metabolite concentrations in the dorsal anterior cingulate cortex (dACC) and bilateral hippocampus were measured by structural MRI and ^1^H-MRS at baseline and after 12 weeks of training. Metabolites measured included glutamate-glutamine (Glx), choline-containing compounds (Cho, including glycerophosphocholine and phosphocholine), gamma-aminobutyric acid (GABA), and N-acetyl aspartate and N-acetylaspartyl-glutamate (NAA-NAAG). In total, 11 participants completed MET and 14 completed yogic meditation for this study. Structural MRI analysis showed an interaction between time and group in dACC, indicating a trend towards increased gray matter volume after the MET intervention. ^1^H-MRS analysis showed an interaction between time and group in choline-containing compounds in bilateral hippocampus, induced by significant decreases after the MET intervention. Though preliminary, our results suggest that memory training induces structural and neurochemical plasticity in seniors with MCI. Further research is needed to determine whether mind-body interventions like yoga yield similar neuroplastic changes.

## Introduction

The global population is aging at a rate unprecedented in human history. About 98 million people will be over 65 years old by 2060 (Mather et al., 2015), which will be paralleled by increased age-related health issues. As a result of the expanding elderly population, cognitive decline and conversion to dementia is becoming an increasingly difficult public health challenge due to high cost of care, morbidity and mortality in patients with Alzheimer’s disease (AD; Alzheimer’s Association, 2015) and age-related dementia (Hurd et al., 2013). Both neuropsychological and neuroimaging studies have suggested that mild cognitive impairment (MCI) represents a prodromal state leading to degenerative dementias including AD (Petersen et al., 2001; Palmer et al., 2002). Therefore, providing interventions to prevent age-related cognitive decline that are cost-effective and easily accessible may prove an effective way to ensure that both patients and caregivers have better quality of life, while reducing financial burden for families and society.

To date, preventive measures against cognitive decline have mainly focused on pharmacotherapy (Karakaya et al., 2013) and physical exercise (Smith et al., 2010; Behrman and Ebmeier, 2014). Behavioral memory training is also popular, based on the notion that cognition is plastic in older age (Acevedo and Loewenstein, 2007; Eyre et al., 2016). For example, traditional memory training interventions that teach mnemonic techniques involving verbal association and visual imagery and practical strategies have been shown to boost cognitive performance, memory, and quality of life in healthy older adults (Verhaeghen et al., 1992; Jean et al., 2010). Given the growing popularity of online “Brain Training” programs, clearer understanding of behavioral memory training programs already demonstrated to be effective in the clinic is needed.

In recent years, mind-body therapies have also been studied as potential preventive measures for MCI (Grossman et al., 2004). By simultaneously targeting multiple physiological and cognitive processes, as well as their dynamic integration, meditation may offer a more efficient alternative to other behavioral interventions. Indeed, some studies indicate that senior meditators have better memory, perceptual speed, attention and executive functioning compared with non-meditators (Prakash et al., 2012), though results are mixed (Chiesa et al., 2011; Goyal et al., 2014). A combination of Kirtan Kriya (KK) meditation and Kundalini Yoga (KY), as used as an intervention in the current study, is specifically shown to affect physical and mental health outcomes (Shannahoff-Khalsa, 2004; Krisanaprakornkit et al., 2006), including older adults with memory complaints (Moss et al., 2012). Like other forms of mind-body practice, KY and KK have been demonstrated to benefit cognitive function, depressed mood and anxiety, sleep and coping (Black et al., 2013; Lavretsky et al., 2013), including older adults with cognitive impairments (Newberg et al., 2010). Thus, given their effectiveness and ease of use, mind-body interventions like KY and KK may be useful tools that can be readily incorporated into future clinical practice.

Despite their promise, the neurobiological mechanisms of behavioral interventions to prevent age-related cognitive decline are not well understood. In the current study, we explored how memory enhancement training (MET) and KY + KK yoga affect brain plasticity in two areas of the brain important for memory and cognition, the bilateral hippocampus and dorsal anterior cingulate cortex (dACC). We used structural magnetic resonance imaging (s-MRI) to measure gray-matter volume and proton magnetic resonance spectroscopy (^1^H-MRS) to measure brain metabolites. We targeted four metabolite peaks with ^1^H-MRS previously linked to cognitive function (Friedman et al., 1998; Rae et al., 1998; Ross and Sachdev, 2004; Stone, 2009; Yoon et al., 2009), specifically: gamma-aminobutyric acid (GABA) and, Glutamate + Glutamine (Glx), which reflect levels of the major inhibitory and excitatory neurotransmitters, respectively (Maddock and Buonocore, 2012; Ramadan et al., 2013); glycerophosphocholine + phosphocholine (Cho), a marker of cell membrane synthesis and breakdown (Maddock and Buonocore, 2012); and N-acetyl aspartate and N-acetylaspartyl-glutamate (NAA/NAAG), which is thought to be a marker of neuronal and axonal viability and density (Ross and Sachdev, 2004).

To our knowledge, this project is the first to compare the effects of MET and KK + KY on brain structure and neurochemistry in healthy seniors with MCI. Notably, our previous study in this cohort demonstrated that improved verbal memory performance in both MET and KK + KY interventions associated with functional neuroplasticity, suggesting yoga and MET may have similar influences on brain function (Eyre et al., 2016). Based on this and related literature indicating reduced brain volume with age and memory disorders (Salat et al., 2004; Lemaitre et al., 2012), we hypothesized that the volume of dACC and bilateral hippocampus would increase after both interventions. Further, we expected that ^1^H-MRS metabolites previously linked with aging and/or memory complaints like choline and NAA/NAAG (Pfefferbaum et al., 1999; Rose et al., 1999) might also change with our interventions. The current study provides further insight into our understanding of the neuroplastic effects of the two interventions, which may help inform future improved, personalized intervention strategies to prevent age-related memory problems and cognitive decline.

## Materials and Methods

### Participants

Participants with subjective memory complaints were recruited via advertisements from UCLA outpatient clinics and UCLA Longevity Center Program from 2014 to 2015. Inclusion criteria included: (1) age ≥55 years; (2) a diagnosis of MCI-amnestic type by standard criteria (Petersen, 2004; Winblad et al., 2004) established at a diagnostic consensus conference; (3) Clinical Dementia Rating scale (Morris, 1993) score of 0.5; (4), sufficient English proficiency at the eighth grade or higher reading level as determined by the word reading subtest of the Wide Range Achievement Test-4 (Wilkinson and Robertson, 2006) to participate in MET; (5) capacity to provide informed consent. Exclusion criteria included: (1) current or past Axis I psychiatric disorders, or recent unstable medical or neurological disorders; (2) any disabilities preventing participation in the MET or KY + KK conditions (e.g., severe visual or hearing impairment); (3) insufficient English proficiency; (4) a diagnosis of dementia per the DSM-IV; (5) Mini Mental Health Examination (Folstein et al., 1975) score of 24 or below; (6) psychoactive medications; (7) participation in psychotherapy involving cognitive training. This study was approved by the UCLA Institutional Review Board (IRB). All participants underwent IRB-approved informed consent procedures prior to enrolling in the study.

### Clinical Measures

Participants were assessed at baseline and at 12-weeks (after training). Clinical measurements included the Geriatric Depression Scale (GDS; Yesavage et al., 1983), Cardiovascular Risk Factors (CVRF; Truelsen et al., 1994), and Mini-Mental State Examination (MMSE; Folstein et al., 1975).

### Yoga and MET Interventions

Participants were randomly assigned to either a 60-min weekly MET or KY + KK (yoga) classes. Each group was assigned approximately 12 min of daily homework specific to the intervention. In total, 25 participants (11 MET and 14 yogic meditation) finished the study.

Memory training (MET) was developed from evidence-based techniques that involve teaching verbal and visual association strategies and other practical strategies to improve memory function. We chose to implement mnemonic methods over computerized memory training programs because the former have a longer history of implementation in aging populations, have been studied more, with much empirical evidence supporting their use in elders.

The KY + KK intervention was conducted by a certified yoga teacher. The KY + KK class included teaching body-movement and mindfulness exercises, specifically: (1) Tuning in (5 min); (2) Warm up (10 min); (3) Pranayam (10 min); (4) Kriya (20 min); (5) Meditation (11 min); and (6) Shavasana (4 min). This standard 12-min KY + KK meditation protocol as created by Yogi Bhajan was taught by the certified yoga teacher. Adverse events were monitored at each visit by using the UKU Side Effect Rating Scale (Lingjaerde et al., 1987).

### MRI Acquisition

**Figure 1:**
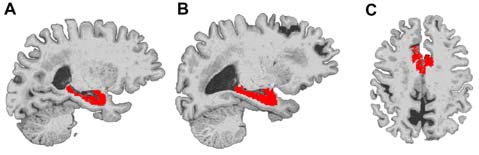
**Example locations of bilateral hippocampus and dorsal anterior cingulate cortex (dACC) regions used in Freesurfer volume analyses.** The right hippocampus **(A)**, left hippocampus **(B)** and dorsal ACC **(C)** are displayed in red on a representative subject’s brain image.

**Figure 2:**
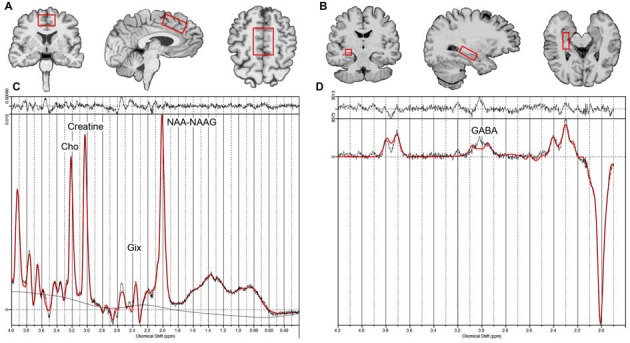
**Example voxel placement for the dorsal ACC (A)**, right hippocampus (**B**, voxel location for the left hippocampus is similar to the right) and example LC model processing output for the non-edited **(C)** and edited difference spectra **(D)**. Creatine, Phosphocreatine + Creatine; Glx, glutamate+ glutamine; Cho, glycerophosphocholine + phosphocholine; NAA-NAAG, N-acetyl aspartate + N-acetylaspartyl-glutamate; GABA, γ-Aminobutyric acid.

Structural MRI (Figure 1) and ^1^H-MRS data (Figure 2) were acquired on a 3.0 Tesla TIM Trio scanner (Siemens, Germany) using a 32-channel head coil, and head motion was minimized with the adjacent placement of firm cushions. High resolution motion-corrected multi-echo MPRAGE images were acquired with the following parameters: TR = 2150 ms, TEs = 1.74/3.6/5.46/7.32 ms, TI = 1260 ms, FA = 7°, FOV = 256 mm × 256 mm, 176 sagittal slices, voxel resolution 1.0 × 1.0 × 1.0 mm^3^. These MRI structural images were resliced to position ^1^H-MRS voxels in dACC (40 × 30 × 20 mm^3^) and bilateral hippocampal gray matter (30 × 12 × 12 mm^3^; see Figures 2A,B). For the hippocampi, single-voxel point resolved spectroscopy (PRESS) sequences were acquired with the following parameters: TR = 2200 ms, spectral bandwidth = 1500 Hz, TE = 30 ms, and 2048 samples with water suppression yielding 128 averages. For the dACC, a J-edited Mescher-Garwood (MEGA) PRESS sequence available as a Siemens Work-in-progress (WIP) was used (Mescher et al., 1996, 1998), which allowed the detection of GABA concentration with spectral editing: TR = 2000 ms, spectral bandwidth = 1500 Hz, TE = 68 ms, and 2048 samples with water suppression yielding 128 averages.

### MRI Structure Preprocessing

FreeSurfer (version 5.1.0, https://surfer.nmr.mgh.harvard.edu/) was used for automated cortical surface reconstruction and cortical/subcortical parcellation estimation on each subject’s structural image. Freesurfer morphometric procedures have been demonstrated to show good test-retest reliability across scanner manufacturers and across field strengths (Han et al., 2006; Reuter et al., 2012). The preprocessing outputs were reviewed, manually corrected and reran as necessary by an experienced MRI analyst. The Desikan-Killiany cortical and FreeSurfer subcortical segmentation atlas was used to estimate volume in regions of interest (ROIs; Fischl et al., 2002, 2004) including the bilateral hippocampus and dACC. Total intracranial volume was estimated for inclusion as a covariate in s-MRI statistical analyses.

### ^1^H-MRS Preprocessing

^1^H-MRS metabolite levels were quantified using the LCModel analysis program (Version 6.3; Provencher, 1993; See Figures 2C,D). The MEGA-PRESS sequence acquires two acquisitions, one with the inversion of GABA multiplet at 1.9 ppm and the other without inversion, which allows GABA J-evolution. GABA is detected by subtracting these two spectra. All other metabolites were estimated using non-edited spectra from either the standard PRESS (for hippocampal voxels) or MEGA-PRESS (for dACC) sequences. Basis sets included of NAA-NAAG, Cho, Glx for one basis file, and GABA for another basis file as suggested by LCModel processing. GABA is reported as GABA-to-NAA/NAAG ratio; remaining metabolites are reported as metabolite-to-creatine ratios.

Cramer-Rao metrics were used for quality control. For Glx, metabolite concentrations from data points with Cramer-Rao lower bounds equal to or exceeding 20% were excluded from analysis. For NAA-NAAG and Cho, Cramer-Rao lower bounds equal to or exceeding 10% were excluded as these metabolites typically display lower fitting error values. These quality-control criteria led to the exclusions listed below; signal-to-noise (SNR) and full width at half-maximum (FWHM) values are also listed for reference for each metabolite. In dorsal ACC: for GABA, 1 Yoga baseline, 3 Yoga follow-up, 3 MET baseline, 1 MET follow-up were excluded (SNR Mean ± SD = 22.21 ± 9.25, FWHM Mean ± SD = 0.05 ± 0.01); for Glx, 1 Yoga baseline, 2 Yoga follow-up, 3 MET baseline, 1 MET follow-up were excluded (SNR Mean ± SD = 24.72 ± 11.63, FWHM Mean ± SD = 0.06 ± 0.02); for Cho, 1 Yoga follow-up, 2 MET baseline, 1 MET follow-up were excluded (SNR Mean ± SD = 23.65 ± 11.97, FWHM Mean ± SD = 0.06 ± 0.02); NAA/NAAG 1 follow-up, 2 MET baseline, 1 MET follow-up were excluded (SNR Mean ± SD = 23.65 ± 11.97, FWHM Mean ± SD = 0.06 ± 0.02). In bilateral hippocampus: for Glx, 1 Yoga baseline, 1 follow-up, 1 MET follow-up were excluded (SNR Mean ± SD = 8.36 ± 2.46, FWHM Mean ± SD = 0.09 ± 0.03); for NAA/NAAG 2 Yoga baseline, 1 Yoga follow-up were excluded (SNR Mean ± SD = 8.42 ± 2.42, FWHM Mean ± SD = 0.09 ± 0.03), for Cho (SNR Mean ± SD = 8.23 ± 2.47, FWHM Mean ± SD = 0.09 ± 0.04).

### Statistical Analysis

For all demographic and clinical measures, the two interventions were compared at baseline by *t*-tests or chi-squared tests. All structural measures and metabolites of interest were analyzed by a mixed effects general linear model (implemented in SAS PROC MIXED), using intervention group, time and the interaction term between time and group as predictors. In analyses of gray-matter volume, total intracranial volume was entered as an additional covariate, and in analyses of hippocampal volume and metabolites, both ROIs were analyzed in the same model with hemisphere (i.e., right and left) entered as an additional within subject factor. *Post hoc* analyses tested within group changes as well as changes between groups with time. As this is an exploratory study to examine how brain structures and metabolites change due to the interventions, we did not correct for multiple comparisons and significance levels were set at 0.05, two-tailed.

## Results

### Demographic, Clinical and Cognitive Measurements

Table 1 presents the baseline clinical and demographic characteristics of study participants. The two groups did not differ significantly on age, sex, race, handedness, education, Body Mass Index (BMI), MMSE, GDS or CVRF at baseline (all *p* > 0.2).

**Table 1:** **Demographic and clinic measures for yoga and memory enhancement training (MET) groups**.

	Yoga group (*n* = 14)	MET group (*n* = 11)
Age (years)	67.1 (9.5)	67.8 (9.7)
Sex (Female)	6 (42.9%)	6 (54.5%)
Race (Caucasian)	12 (85.7%)	8 (72.7%)
Handedness (Right)	12 (85.7%)	8 (72.7%)
Education	16.8 (1.7)	16.2 (1.6)
BMI	26.7 (4.3)	24.5 (3.6)
MMSE	28.7 (1.3)	29.1 (0.7)
GDS	7.5 (5.1)	5.7 (5.6)
CVRF	8.3 (5.2)	6.6 (4.8)

*Figures indicate mean with standard deviation (SD), or number with of subjects with percentage (%) in parentheses. Abbreviations: Education, years of education; BMI, Body Mass Index; MMSE, Mini-Mental Status Exam; GDS, Geriatric Depression Scale; CVRF, Cardiovascular Risk Factor*.

### MRI Structural Analysis

We examined gray-matter volume changes after yoga and memory training in bilateral hippocampus and dACC (Table 2 and Figure 1). An effect size (between group Cohen’s *d*) for gray-matter volume changes (see Table 2) was also calculated. Analyses for the hippocampus did not reveal a significant interaction term between time and group (*F*_(1,23)_ = 1.35, *p* = 0.25) indicating that change in hippocampal volume did not differ between the two interventions. In dACC, there was a significant interaction term (*F*_(1,22)_ = 4.86, *p* = 0.04); the memory training group showed a trend towards increased volume after training (*t*_(22)_ = 1.73, *p* = 0.1) while the yoga group did not show any change (*t*_(22)_ = 1.38, *p* = 0.2). Further, there were no significant main effects of group or time for either region.

**Table 2:** **Magnetic resonance imaging (MRI) volumes for yoga and MET groups**.

Region	Yoga group Mean (SD) (mm^3^)		MET group Mean (SD) (mm^3^)	
	Baseline	Follow-up	Baseline	Follow-up	Time*group	Effect size
Dorsal anterior cingulate cortex	2040.8 (363.3)	2022.0 (319.2)	1677.4 (239.6)	1716.1 (239.9)	*F*_(1,23)_ = 4.9, *P* = 0.04	0.72
Bilateral hippocampus	3492.7 (477.4)	3508.6 (465.4)	3397.2 (321.1)	3438.9 (436.5)	*F*_(1,23)_ = 1.4, *P* = 0.2	0.08

*The reported effect sizes are the between group Cohen’s d for changes in MRI volumes*.

### ^1^H-MRS Brain Metabolite Analysis

For ^1^H-MRS analyses, we analyzed Glx, NAA/NAAG, and Cho values, as well as the effect size for the comparisons in bilateral hippocampus and dACC. GABA was also estimated in the dACC using spectral editing as described above (see Table 3). In bilateral hippocampus, a significant interaction between time and group was found for choline (*F*_(1,38)_ = 4.35, *p* = 0.04; See Table 3 and Figure 2). *Post hoc* analyses showed that choline decreased in the memory training group (*t*_(38)_ = −2.58, *p* = 0.01), but did not change in the yoga group (*t*_(38)_ = 0.30, *p* = 0.8). Of note, though all other measures were similar across treatment groups, hippocampal choline was greater in the memory training group at baseline than the yoga group (*t*_(38)_ = 2.17, *p* = 0.04). No other metabolites exhibited significant interaction terms, nor were there significant main effects of group or time for any metabolite for the hippocampus. In the dACC, there were no significant interactions or main effects identified for any metabolites.

**Table 3:** **Proton magnetic resonance spectroscopy (^1^H-MRS) signal intensity ratio measures for yoga and MET groups**.

	Yoga group Mean (SD)		MET group Mean (SD)	
	Baseline	Follow-up	Baseline	Follow-up	Time*group	Effect size
Dorsal ACC
GABA	0.35 (0.18)	0.33 (0.17)	0.35 (0.20)	0.31 (0.14)	*F*_(1,17)_ = 0.07, *P* = 0.8	0.44
Glx	0.74 (0.22)	0.94 (0.48)	0.99 (0.59)	1.26 (1.24)	*F*_(1,17)_ = 0.67, *P* = 0.4	0.62
Cho	0.27 (0.07)	0.26 (0.04)	0.25 (0.03)	0.26 (0.05)	*F*_(1,19)_ = 0.51, *P* = 0.5	0.41
NAA/NAAG	1.21(0.17)	1.24 (0.18)	1.30 (0.15)	1.28 (0.11)	*F*_(1,19)_ = 0.24, *P* = 0.6	0.06
Bilateral hippocampus
Glx	1.77 (0.35)	1.83 (0.37)	1.72 (0.35)	1.88 (0.46)	*F*_(1,40)_ = 0.33, *P* = 0.6	0.18
Cho	0.31 (0.04)	0.32 (0.03)	0.34 (0.05)	0.31 (0.04)	*F*_(1,38)_ = 4.35, *P* = 0.04	0.42
NAA/NAAG	1.17 (0.11)	1.10 (0.12)	1.14 (0.13)	1.17 (0.19)	*F*_(1,40)_ = 2.90, *P* = 0.09	1.02

*The reported effect sizes are the between group Cohen’s d for changes in MRS signal intensity measures. Signal intensity measures were obtained by the ratio of creatine and phosphocreatine (Cr + Pcr) except gamma-aminobutyric acid (GABA) by the ratio of N-acetyl aspartate and N-acetylaspartyl-glutamate (NAA/NAAG). Abbreviations: Dorsal ACC, Dorsal anterior cingulate cortex; Gix, Glutamate + Glutamine; Cho, glycerophosphocholine + phosphocholine*.

### Correlation Analysis Between MRS, MRI and Clinical Measurements

We explored whether the changes for clinical measures correlated with the structural or brain chemical changes. None of the correlations between the MRI or MRS changes and GDS or MMSE changes were significant. Note also that followup measures were also reported previously (Eyre et al., 2016); after interventions, GDS Mean(SD) was 3.9(2.5) for Yoga Group and 3.2(2.5) for MET group, and MMSE was 29.0(0.9) for Yoga Group, 29.0(1.0) for MET group.

## Discussion

Taken together, our results suggest that behavioral interventions like MET can impact the anatomy and neurochemistry of the aging brain. In MRI analyses, we found that the MET intervention was associated with decreased choline levels in bilateral hippocampus, as well as modest, but significant increases in gray-matter volume in dACC. This is the first pilot study to evaluate structural and metabolite neuroplasticity associated with MET and yoga interventions in seniors with MCI using ^1^H-MRS and s-MRI techniques to our knowledge. However, given our relatively small sample size and the exploratory nature of the study, future research is needed to validate these results, and to determine whether yoga can induce similar patterns of neuroplasticity as we have demonstrated previously with functional MRI (Eyre et al., 2016).

### Implications for Increased Hippocampal Choline in Cognitive Aging

^1^H-MRS is potentially a powerful tool, offering a non-invasive approach to measuring brain metabolites *in vivo*. In our data, we found that choline in bilateral hippocampus decreased after MET training in seniors with MCI. Choline is a common target in ^1^H-MRS studies, and its peak reflects the presence of phosphorylcholine and glycerophosphoryl-choline. These choline-containing compounds play significant roles in synthesis and breakdown of lipid components of cell membranes, and as precursors for the neurotransmitter acetylcholine (Amenta and Tayebati, 2008). Choline levels measured with ^1^H-MRS have been demonstrated to increase in the aging brain (Pfefferbaum et al., 1999; Haga et al., 2009) and in AD (Pfefferbaum et al., 1999; Kantarci et al., 2000, 2007). Specifically, regarding ^1^H-MRS in the hippocampus and medial temporal cortex, studies have reported increased choline with age in humans (Angelie et al., 2001) and in animal models (Katz-Brull et al., 2002), though results are mixed (Haga et al., 2009) and hippocampal choline may not be elevated in MCI when compared to healthy seniors (Tumati et al., 2013). However, studies using more sensitive and invasive techniques to measure choline are more consistent; choline and choline-containing compounds measured directly from CSF via lumbar puncture seem to be consistently elevated in AD (Elble et al., 1989; Walter et al., 2004; Ibáñez et al., 2012) and MCI (Ibáñez et al., 2012). Thus, our results may suggest that behavioral interventions like MET can reduce age-related choline increases in the hippocampus. The mechanisms underlying these effects cannot be addressed directly with MRI; however, future studies will be better able to determine, for example, whether these changes in choline-containing compounds relate to membrane turnover, acetylcholine synthesis, or other factors (Ibáñez et al., 2012).

Medial temporal lobe structures like the hippocampus are critical brain regions involved in memory (Tulving and Markowitsch, 1998). Hippocampal volume is often linked to performance on memory tasks (Schultz et al., 2015), and has also been consistently demonstrated to change with memory training and intervention (Erickson et al., 2011). Hippocampal volume is also known to decrease with age and in association with MCI, AD and other age-related disorders (Lupien et al., 1998; Elcombe et al., 2015). Given that choline-containing compounds measured with ^1^H-MRS are associated with cell-membrane turnover, one might expect corresponding structural changes in the hippocampus as well; however, we did not find a link between hippocampal volume and memory training in the current study. This could be due to our relatively small sample, relatively short training time, and/or other aspects of our methodological approach. For example, some studies have indicated that structural plasticity in the hippocampus might vary across hippocampal subfields in relation to memory interventions (Engvig et al., 2012) and AD status (Mueller et al., 2007). Clearly, more work is needed to determine the relationship between structural plasticity in the hippocampus and behavioral interventions for age-related memory complaints.

### Role of Anterior Cingulate Cortex in Cognitive Aging

In our study, we provide novel evidence that a behavioral memory intervention (MET) can modestly increase cortical gray matter in dACC, a region of the brain linked to multiple key cognitive functions, such as error detection (Gehring et al., 1993), and executive processing (Carter et al., 2000). Gray-matter volume has been demonstrated to decrease with age in the ACC in both cross-sectional (Sowell et al., 2003) and longitudinal studies (Resnick et al., 2003). Correspondingly, age is negatively correlated with blood flow in dorsal and rostral ACC regions (Vaidya et al., 2007). Seniors who engage more in cognitive games and puzzles in their daily lives also tend to have greater ACC gray matter volume (Schultz et al., 2015), which is consistent with our results and raises the possibility that engaging in cognitive-behavioral games or training could prevent age-related structural atrophy in this region. Indeed, a recent study indicated a trend towards increased rostral ACC thickness in seniors after MET; however, this effect did not survive a stringent validation analysis (Engvig et al., 2010). Although our effects are modest, they do indicate that participating in effective behavioral interventions may help to ameliorate age-related brain changes associated with poor memory and cognitive performance.

### Yoga and the Aging Brain

Structural plasticity in the dACC and hippocampus has also been associated with yoga practice in previous studies; however, we did not find evidence of gray-matter volume changes in dACC or hippocampus after our 12-week yoga intervention. Yoga has been linked to anatomical changes in frontal cortex (Baijal and Srinivasan, 2010; Froeliger et al., 2012; Villemure et al., 2014; Desai et al., 2015), anterior cingulate cortex (ACC) and insula (Nakata et al., 2014; Villemure et al., 2014, 2015), and the hippocampus (Froeliger et al., 2012; Villemure et al., 2015). However, many of these studies compare the brains of practiced yogis with several months or years of experience to yoga-naive controls (Froeliger et al., 2012); perhaps the relatively shorter length of training in the current study (12 weeks) was less conducive to detecting structural plasticity associated with our yoga intervention. In this same cohort, we have already demonstrated that memory improvements after yoga and MET may induce functional plasticity in similar brain regions (Eyre et al., 2016).

### Limitations

As with all research, there are limitations one should consider when interpreting the results of the current study. Our relatively small sample size and short duration of yogic meditation and memory trainings may have compromised our power to detect significant effects. This may have also played a role in our finding indicating that the yoga and MET groups differed in hippocampal choline at baseline; incidental findings like these are a challenge accompanying pilot studies like ours. Future studies (Flak et al., 2014) with larger sample sizes will provide critical independent validation of our results. However, despite these limitations we did detect two significant and potentially biologically meaningful effects, which may be generalizable to a broad population of seniors with MCI given that both intervention groups were matched for demographic features. The current study did not include a placebo or period without an intervention as a control condition, or a group of healthy seniors without MCI, which means that we are unable to directly compare our findings with neuroplasticity that might occur with typical aging similar time windows (i.e., 12 weeks). We also targeted structural and metabolite changes using ^1^H-MRS and s-MRI; future studies integrating across more and different MRI modalities may be better able to examine different aspects of brain plasticity occurring after behavioral interventions like yoga and MET. Notably, GABA, which has a relatively low concentration in the brain, was only sampled in the dACC given that the voxel size for the hippocampus was considered too small to achieve the required SNR. In spite of these possible limitations, this study is a promising start to elucidating the brain mechanisms of behavioral interventions for seniors with MCI.

### Conclusion

The present study examined changes in brain metabolites and structure among individuals undergoing memory training and yogic meditation. We demonstrated that memory training over 3 months is associated with decreased choline levels in bilateral hippocampus and increased gray-matter volume in dACC, suggesting that behavioral interventions like MET may ameliorate markers of brain aging. These effects are somewhat modest, and would benefit from independent validation in larger samples and perhaps over longer-duration interventions. However, these findings suggest that engaging in cognitive activities and mind-body practices may affect the brain in positive ways, and may be combined as part of a multi-faceted approach to encourage healthy aging.

## Author Contributions

HL, KLN, DSK, LE and PS designed the research; HY, NMSC and HL performed the research; HY, AML and PS analyzed the data; and HY, AML, PS, PP, HAE, KLN and HL wrote the article.

## Funding

This study was supported by the Alzheimer’s Research and Prevention Foundation (ARPF), and 2014 National Alliance for Research on Schizophrenia and Depression (NARSAD) Young Investigator Grant from the Brain and Behavior Research Foundation (22325).

## Conflict of Interest Statement

The authors declare that the research was conducted in the absence of any commercial or financial relationships that could be construed as a potential conflict of interest. The reviewer AJW and handling Editor declared their shared affiliation, and the handling Editor states that the process nevertheless met the standards of a fair and objective review.
